# Evaluation of Cotton Fleahopper (*Pseudatomoscelis seriatus* (Reuter)) Feeding on Mpp51Aa2-Traited Cotton Utilizing Electrical Penetration Graph (EPG) Waveforms

**DOI:** 10.3390/insects15050316

**Published:** 2024-04-29

**Authors:** Brady P. Arthur, Charles P.-C. Suh, Benjamin M. McKnight, Megha N. Parajulee, Fei Yang, Thomas M. Chappell, David L. Kerns

**Affiliations:** 1 Department of Entomology, Texas A&M University, College Station, TX 77843, USA; bparthur@tamu.edu; 2 USDA-ARS Southern Plains Agricultural Research Center, College Station, TX 77845, USA; 3 Department of Soil and Crop Sciences, Texas A&M University, College Station, TX 77843, USA; 4 AgriLife Research and Extension Center, Texas A&M University, Lubbock, TX 79403, USA; 5 Department of Entomology, University of Minnesota, St. Paul, MN 55108, USA; 6 Department of Plant Pathology and Microbiology, Texas A&M University, College Station, TX 77843, USA

**Keywords:** Mpp51Aa2, ThryvOn, cotton fleahopper, *Psuedatomoscelis seriatus*, *Gossypium hirsutum*, electropenetrography, electrical penetration graph, EPG

## Abstract

**Simple Summary:**

Historically, the pesticidal *Bacillus thuringiensis* proteins in transgenic cotton cultivars have shown no activity on mirid pests such as the cotton fleahopper. Therefore, control options are limited to foliar applied insecticides, which are often not optimized for reducing potential yield losses. With the inclusion of the Mpp51Aa2 protein, termed ThryvOn, damage from cotton fleahopper feeding can be mitigated. While the effect of the protein has been observed in field studies, there is a limited understanding of the interaction of the protein and the insect. Therefore, the focus of this study was the use of electropenetrography to monitor the feeding behaviors of the cotton fleahopper on both ThryvOn and a non-ThryvOn cotton cultivars. We found that although attempts were made by insects to feed on ThryvOn cotton, they exhibited shorter durations of plant fluid ingestion when feeding. Additionally, the percentage of ingestion events per insect that were sustained was lower. These findings indicate potential damage to the insect gut, consistent with the known symptoms associated with the activity of Bt proteins.

**Abstract:**

Prior to the recent implementation of the Mpp51Aa2 pesticidal protein (ThryvOn), transgenic cotton cultivars have historically offered no control of the cotton fleahopper (*Pseudatomocelis seriatus* (Reuter)). To evaluate the feeding behavior of cotton fleahoppers on ThryvOn cotton, electropenetrography (EPG) using a Giga-8 DC instrument was used to monitor the probing activity of fourth- and fifth-instar cotton fleahopper nymphs on both ThryvOn and non-ThryvOn cotton squares. Nymphs were individually placed on an excised cotton square for 8 h of EPG recording, after which resulting waveforms were classified as non-probing, cell rupturing, or ingestion. Although there were significantly more cell rupturing events per insect on ThryvOn (mean ± SEM, 14.8 ± 1.7) than on non-ThryvOn squares (mean ± SEM, 10.3 ± 1.6), there was no difference attributable to ThryvOn in the average number of ingestion events per insect. However, the average duration of ingestion events was significantly shorter on squares with ThryvOn (mean ± SEM, 509 ± 148 s) than on squares without (mean ± SEM, 914 ± 135 s). This suggests that cotton fleahoppers continued to probe despite their inability to sustain ingestion. These results provide conclusive evidence that the Mpp51Aa2 pesticidal protein affects the feeding behavior of cotton fleahopper nymphs.

## 1. Introduction

With the success of the boll weevil (*Anthonomus grandis grandis* (Boheman)) eradication program and the wide adoption of transgenic cultivars targeting lepidopteran cotton pests, there has been a substantial reduction in broad-spectrum insecticide applications used to control these in-season pests [1,2]. This has prompted a rise in the economic impact of the cotton fleahopper (*Pseudatomoscelis seriatus* Reuter) (Hemiptera: Miridae), which now ranks as one of the most detrimental cotton insect pests in Texas, USA. The prevalence of cotton fleahopper infestations has risen significantly in Texas from 30% of total cotton acres in 2017 to an estimated 93% of cotton acres in 2022 [3,4]. Selection for naturally occurring resistance traits has shown some promise but has not had a consistent level of success. Therefore, foliar insecticide applications remain the most effective way to manage cotton fleahopper infestations [5,6]. Often, multiple foliar insecticide applications are needed during the growing season, and the timeliness of applications can be critical [7]. However, the continued use of broad-spectrum foliar insecticides is detrimental from both an economic and environmental perspective because applications are costly and populations of natural enemies may be significantly reduced [4,6]. This has prompted a shift toward transgenic traits offering protection against the feeding of piercing–sucking insect pests such as the cotton fleahopper.

Historically, the Cry proteins that have been incorporated and expressed by commercial cotton cultivars have no efficacy on hemipterans [8]. The introduction of the Mpp51Aa2 protein, also referred to as Cry51Aa2 [9], in transgenic cotton cultivars by Bayer CropScience has resulted in the first cotton cultivars with confirmed activity on hemipteran and thysanopteran pests in cotton [10,11]. Like other Cry proteins, the Mpp51Aa2 pesticidal protein targets the epithelial cells in the insect midgut, resulting in pore formation [10,11]. Activity on the tarnished plant bug has been confirmed by Jerga et al. [11], where dissected midguts of tarnished plant bug nymphs (*Lygus lineolaris* Palisor de Beauvois) showed significant damage associated with the activity of pesticidal proteins. The efficacy of the Mpp51Aa2 protein on mirid species has been confirmed using artificial diet studies, where the lethal concentrations (LC_50_) for tarnished plant bugs and the western tarnished plant bugs (*Lygus hesperus* Knight) were 0.853 µg mg^−1^ and 0.3 µg mg^−1^, respectively [10]. Furthermore, the concentration of Mpp51Aa2 has been shown to correlate positively with nymph mortality and negatively with nymph mass [12].

Field trials evaluating cotton expressing Mpp51Aa2, henceforth referred to as ThryvOn, have shown inconsistent results in reducing populations of mirid pest species. Some field evaluations have credited ThryvOn with a reduction in the number of adult cotton fleahoppers [6] and tarnished plant bugs [13] across multiple locations. Conversely, Whitfield [14] and Arthur et al. [15] reported no significant reduction in tarnished plant bug or cotton fleahopper adults but noted lower numbers of small and large nymphs for both pests. Based on a three-year study, Arthur et al. [15] did not find a consistent reduction in cotton fleahopper populations in ThryvOn cotton. However, there were significantly fewer large nymphs found in Mpp51Aa2 cotton compared with the non-ThryvOn control. Despite inconsistent results relating mirid pest population densities to ThryvOn, many studies have shown that ThryvOn cotton offered higher fruit retention compared to non-ThryvOn when exposed to mirid pests in both field studies and laboratory experiments [13,14,15,16]. When integrated into a pest management system, fewer applications of foliar insecticides were needed on ThryvOn cotton than were necessary for non-ThryvOn cotton [14,17].

Like other mirids, cotton fleahoppers have a modified proboscis with no salivary sheath and feed through a “macerate-and-flush” process [18,19]. They rely on their stylets to penetrate plant cells and to secrete lytic enzymes such as polygalacturonates and proteases [20,21,22]. These enzymes initiate the process of digestion by breaking down plant cells and flushing nutrient sap from plant cells to be ingested [23]. The breakdown of plant cells by the salvia also triggers cotton’s production of ethylene, ultimately leading to square abscission [24]. Despite some literature supporting the idea that mechanical damage alone may result in square abscission [25], the salivation phase during mirid feeding is widely considered to be the primary cause of plant injury [26]. Backus et al. [19] termed this process of pre-ingestion plant cell maceration from both mechanical and enzymatic damage “cell rupture feeding”. Recordings of cotton feeding mirid pests, the tarnished plant bug and western tarnished plant bug, have been characterized on cotton plant tissues with discernable waveform patterns of both probing and non-probing behaviors [27,28,29,30]. Because this process has been characterized for other mirid pests and given the similarities in the feeding apparatus and behaviors among mirids, monitoring it in cotton fleahoppers may be conducted similarly.

To monitor the feeding of cotton fleahoppers on plant tissues, electropenetrography (EPG) can be used to describe feeding behaviors based on changes in electrical current flowing through the insect and into the plant where the stylet has penetrated the cells [31]. Electropenetrography records changes in the flow of electrical current that occur when insects insert their proboscis into plant tissue. The variable flow of electrical current from the insect’s mouth parts through the plant substrate is determined by the insect’s behavior and results in recognizable waveforms that can be classified to indicate certain behaviors. Voltage changes are influenced by two primary components, electrical resistance (Ri) and biopotentials (emf) [31]. The R component can be described as the mechanical resistances encountered by ionically charged fluids passing through the insect stylets. This resistance component accounts for the movement of fluids throughout the insect and plant during feeding. Conversely, the emf component reflects changes in biological feeding processes affecting voltages and the biopotentials of the insects nervous system [28]. Identification of the different patterns of voltage changes between biological processes allows for the characterization of waveforms. For instance, when an insect’s stylet penetrates and ruptures plant cells, the resulting voltage changes through time will be discernable from those associated with plant sap ingestion [29].

Given the consistent benefit of increased fruit retention despite inconsistent control of field populations of *Lygus* spp. and cotton fleahoppers in ThryvOn cotton, a more in-depth investigation of the interaction between the ThryvOn trait and target insect is needed to determine how the trait affects the insect’s ability to feed [13,14,15]. Therefore, the primary objective of this study was to use electropenetrography to determine differences in cotton fleahopper feeding behaviors on ThryvOn cotton in comparison to a near isoline that does not express the Mpp51Aa2 protein.

## 2. Materials and Methods

### 2.1. Plant and Insect Sources

Electropenetrography was used to monitor and record the feeding behaviors of cotton fleahopper nymphs on two near isoline cultivars. The selected cultivars included Deltapine 2131 BG3TXF and Deltapine 2038 BG3XF, the first of which expresses the Mpp51Aa2 protein and is termed ThryvOn. Isoline cultivars were selected to ensure they were genetically similar, including the expression of lepidopteran-targeting pesticidal proteins Cry1Ac, Cry2Ab2, and Vip3a19 pesticidal proteins. While these proteins are expressed in both cultivars, they have displayed no activity on hemipterans to date [8]. In 2023, both cultivars were planted in Snook, TX, USA at the Texas A&M University Field Research Farm. The field was managed based on standard local practices as presented by the Texas A&M AgriLife Extension Service [32,33]. No foliar insecticides were applied to plants that were used for electropenetrography monitoring.

Previous work on mirids in cotton has shown that later-instar nymphs are in the more damaging life stage [16,34]. Therefore, only fourth- and fifth-instar cotton fleahopper nymphs were used in our study. Cotton fleahopper nymphs were collected utilizing a beat bucket from a commercial non-ThryvOn cotton field that had not been treated with insecticides near Snook, TX. Nymphs were collected between 0800 and 0900 h each morning before EPG recordings were performed to ensure only lively and vigorous nymphs were used in the study. Approximately one hour after collection, fourth- and fifth-instar nymphs were selected and placed in an environmentally controlled room where the EPG recordings were conducted, with conditions maintained at 25 to 28 °C and relative humidity of 38 to 41%. Lighting in the climate-controlled room remained on for the time the EPG monitoring recordings were in progress.

### 2.2. Insect Wiring and EPG Instrument Configuration

Insects were immobilized by low suction on the ventral side of the thorax under a digital dissecting microscope. An 18 µm gold wire (EPGSystems, Wageningen, The Netherlands), approximately 2.5 cm in length, was glued to the dorsal side of the thorax using conductive silver glue. The silver glue contained white household glue, water, and silver flakes at a ratio of 1:1:1 (*v*:*v*:*w*) (EPGSystems, Wageningen, The Netherlands). Nymphs were then placed in a copper wire Faraday cage and connected to an 8-channel Giga 8-dd DC EPG amplifier (EPGSystems, Wageningen, The Netherlands), where they were suspended from the wire for one hour before being placed individually onto a cotton square (floral bud). While cotton fleahopper damage is most commonly noted on matchhead squares or smaller, they will feed on and damage squares up to 10 mm in diameter [25]. Therefore, larger squares (~8 mm in diameter) were utilized to ensure proper connection with the negative electrode from the Giga-8dd amplifier. Selected squares were excised from the plant, and the copper ground electrode from the Giga 8-dd was inserted into the base of the square and positioned on a plastic platform. For each replicate, four squares from the ThryvOn and four squares from the non-ThryvOn were randomly assigned a channel. Insects were positioned onto the respective squares, and each channel’s waveform was recorded for 8 h. The experiment was repeated for 15 replicates for a total of 60 insects per cultivar. All channels were set to an Ri level of 10^9^ Ω, and the input voltage and gain were individually calibrated based on the methods described by Tjallingii [35]. Signals were recorded at a sample rate of 100 Hz per insect using EPG Stylet+d v01.34 software [36].

### 2.3. Waveform Characterization and Statistical Analyses

Given the biological similarity between the cotton fleahopper other mirids [18]), patterns in feeding waveforms across the species are similar. Thus, waveforms for probing and non-probing behaviors for cotton fleahopper nymphs were characterized utilizing the software Stylet+a v01.30 [37] and the same classifications and definitions used by Cervantes et al. to characterize the feeding behaviors of *L. hesperus* and *L. lineolaris* in cotton [29]. Because the primary objective was to compare the feeding behaviors of the cotton fleahopper nymphs on the two cultivars, non-probing (NP) activities of standing, walking, and antennation were combined for analysis. Similar to the methods of Backus et al. [27], characterization of probing behaviors included ingestion (I) and cell rupturing (CR). Cell rupturing is composed of non-ingestion behaviors associated with probing, which include salivation, tasting, and transitional behaviors [27,29].

Summary statistics selected for comparison included the total duration of non-probing events per insect, mean duration of cell rupturing events, the total duration of all cell rupturing events per insect, mean duration of ingestion events, the total duration of ingestion events per insect, the average number of ingestion events per insect, the average number of cell rupturing events per insect, and the percentage of ingestion events sustained. Comparisons of the feeding behaviors of the cotton fleahoppers between the two cultivars were assessed using a hierarchical ANOVA, in which a replicate (insect recording) was nested within the treatments of ThryvOn and non-ThryvOn. Tests for differences in feeding behaviors between treatments were conducted as *t*-tests with α = 0.05. Regression curves were generated by plotting the relationship of cell rupturing events per insect to the number of ingestion events per insects. Comparison of the linear and non-linear models’ fit to the corresponding regression was conducted by an extra sum-of-squares F-test at α = 0.05. Differences in the percentages of sustained ingestion events on ThryvOn and non-ThryvOn were determined utilizing a chi-square test of independence (α = 0.05). All statistical procedures were completed utilizing Graphpad Prism 9.3.0 [38].

## 3. Results

### 3.1. Waveform Characterization

**Figure 1:**
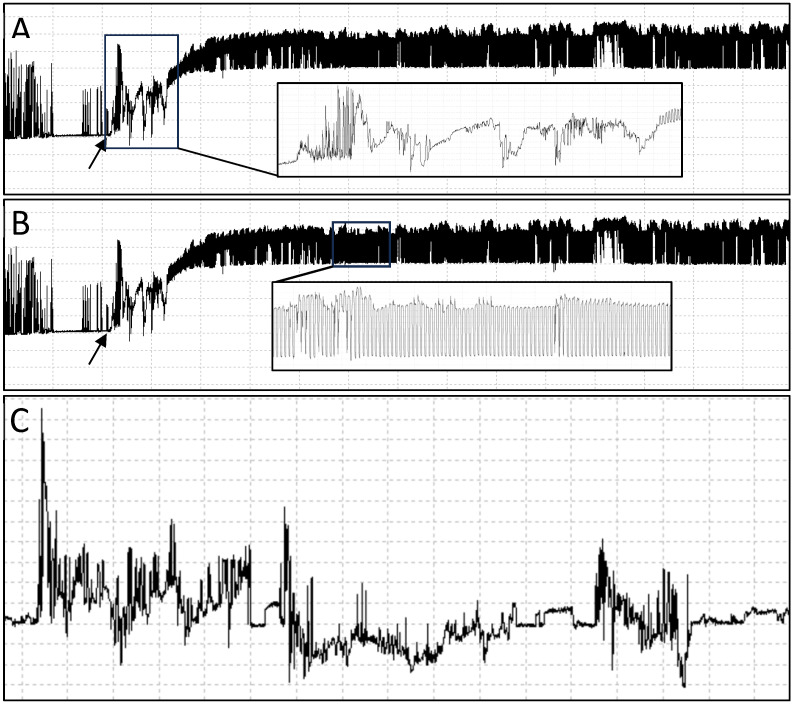
Overview of characteristic electropenetrography waveforms associated with cotton fleahopper probing behaviors: cell rupturing events (**A**), ingestion events (**B**) and non-probing events (**C**). Boxed sections of the waveform are magnified in the inset boxes. Arrows indicate the initiation of a probe.

All nymph waveforms were characterized for non-probing and probing behaviors; however, not all nymphs fed, so only those that initiated a probing behavior during the 8 h recording period were considered when comparing feeding behaviors of cotton fleahopper nymphs on the respective cultivar. Similar to the characterization of *Lygus spp*. from Cervantes et al. [29], probe initiations could be identified by a high amplitude peak followed by the cell rupturing (CR) waveform. Cell rupturing waveforms were irregular in length and magnitude but appeared in each probe (Figure 1A). Ingestion waveforms (Figure 1B) displayed a more rhythmic pattern at relatively high frequencies. Thus, the EPG feeding waveforms we detected for cotton fleahopper were synonymous with those described by Cervantes et al. [29] for *L. lineolaris* and *L. hesperus*. Each period of a particular waveform was characterized as an event. Cell rupturing events always followed the initial peak of probe initiation but also followed an ingestion event on some occasions. In contrast, ingestion events only followed a cell rupturing event, but did not occur after every cell rupturing event. A total of 34 and 41 cotton fleahoppers initiated probing activity on the ThryvOn and non-ThryvOn cultivars, respectively.

### 3.2. Number of Cell Rupturing and Ingestion Events

**Figure 2:**
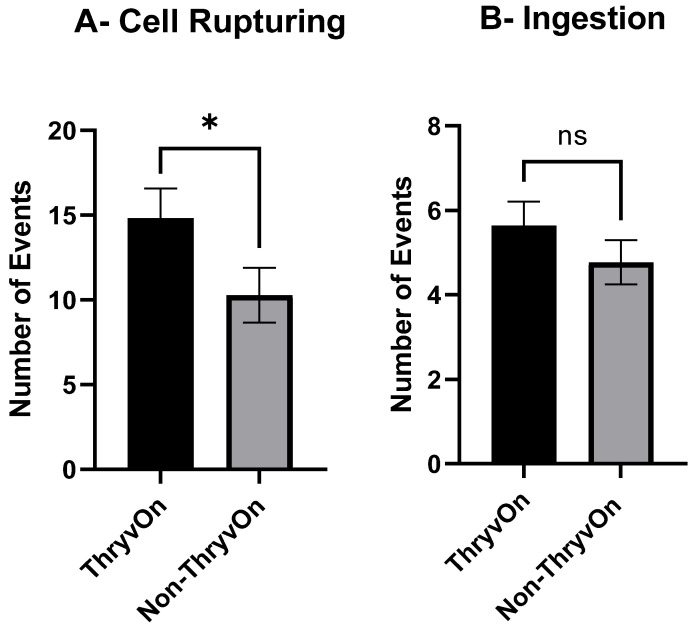
Mean ± SEM numbers of cell rupturing events (**A**) and ingestion events (**B**) recorded for cotton fleahopper nymphs on ThryvOn and non-ThryvOn cotton. Asterisks indicate treatments are significantly different (ANOVA, Student’s *p* < 0.05), ns indicates no significant differences.

The number of cell rupturing and ingestion events per insect were totaled to compare feeding activity on ThryvOn squares and non-ThryvOn squares. During a feeding event, nymphs displayed multiple occurrences of each feeding behavior and often transitioned between them. The mean ± SEM number of cell rupturing events per insect on non-ThryvOn squares was 10.27 ± 1.620 events, which was significantly lower than the 14.82 ± 1.743 events observed on ThryvOn squares (*t*_73_ = 1.912, *p* = 0.0325) (Figure 2A). Conversely, the mean ± SEM number of ingestion events per insect was not significantly different between treatments, averaging 5.77 ± 0.568 and 4.64 ± 0.520 events per insect on ThryvOn and non-ThryvOn, respectively (*t*_73_ = 1.124, *p* = 0.2649) (Figure 2B).

### 3.3. Relationship of Cell Rupturing Events and Ingestion Events

**Figure 3:**
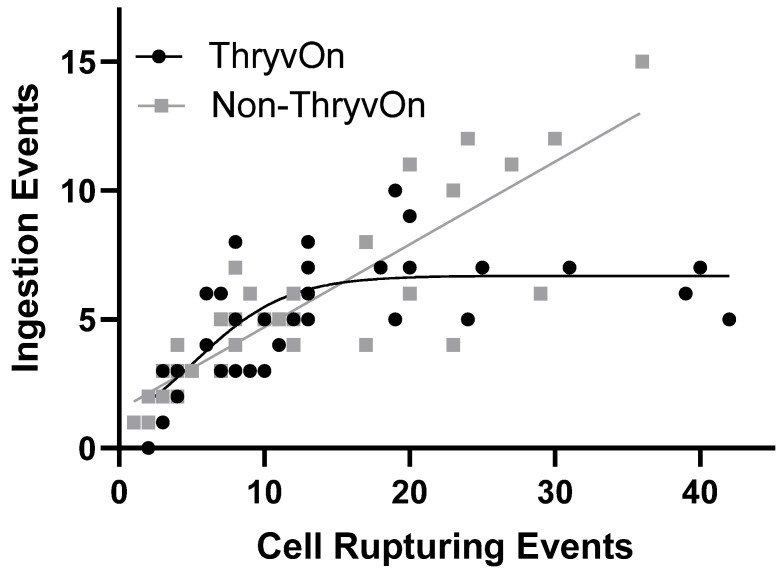
Relationship between cell rupturing events and the number of ingestion events between ThryvOn and non-ThryvOn cotton squares. The relationship of the feeding behaviors on ThryvOn was best fit by a logistic growth regression model (I = 7.381344/(5.582 × 10^(−0.313*CR) + 1.104)^) (R^2^ = 0.5262), while the non-ThryvOn was a linear regression model I = (0.3208 × CR + 1.485) (R^2^ = 0.7641).

Figure 3 shows regressions analyses of cell rupturing events per insect relative to the number of ingestion events per insect for each insect on non-ThryvOn or ThryvOn squares using the best fit model. A linear relationship was determined as the best fit model for non-ThryvOn (*F*_1_ = 0.0221, *p* = 0.8825), but a non-linear logistic model best described the relationship on ThryvOn (*F*_1_ = 17.77, *p* = 0.0002). The linear model for non-ThryvOn squares (I = (0.3208 × CR + 1.485) (R^2^ = 0.7641) indicated a consistent relationship between ingestion and cell rupturing events per insect. However, the logistic model for ThryvOn (I = 7.381344 / (5.582 × e^(−0.313*CR) + 1.104)^) (R^2^ = 0.5262) revealed that the maximum number of ingestion events per insect on ThryvOn squares was 6.68.

### 3.4. Duration of Cell Rupturing Events

**Figure 4:**
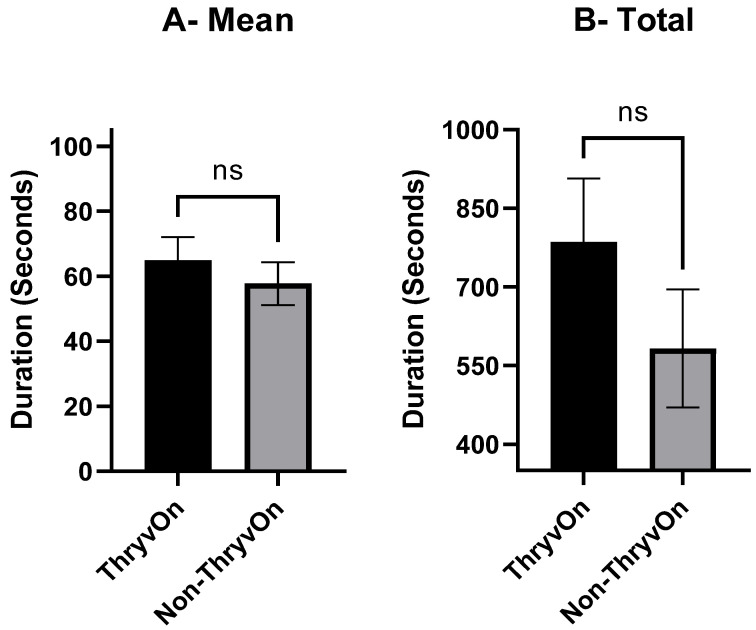
Mean ± SEM duration (**A**) and total duration (**B**) of cell rupturing events exhibited by cotton fleahopper nymphs on ThryvOn and non-ThryvOn cotton. Asterisks indicate treatments are significantly different (ANOVA, Student’s *p* < 0.05), ns indicates no significant differences.

While discernable from ingestion events, cell rupturing events per insect are associated with the probing activity of mirids and consist of several indiscernible actions. These activities include proboscis insertion, salivation, and tasting, all resulting in voltage changes based on the flow of ionically charged fluid flowing through the proboscis and between plant cells as enzymatic plant cell degradation occurs. There was no significant difference between the mean ± SEM duration of a cell rupturing event on ThryvOn (64.99 ± 7.083 s) and non-ThryvOn squares (57.77 ± 6.596 s, ±SEM) (*t*_73_ = 0.7441, *p* = 0.4592) (Figure 4A). To compare the total duration of cell rupturing time, all cell rupturing events for each insect were summed (Figure 4B). The mean ± SEM total duration of cell rupturing events per insect was 786.0 ± 121.007 s for ThryvOn and 582.6 ± 112.671 s for the non-ThryvOn (±SEM), which were not significantly different (*t*_73_ = 1.228, *p* = 0.2236).

### 3.5. Duration of Ingestion Events

**Figure 5:**
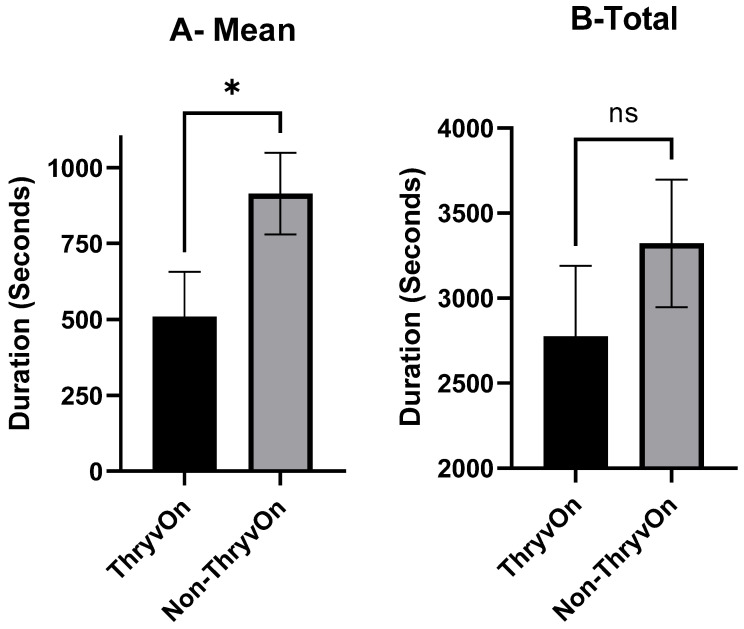
Mean ± SEM duration (**A**) and total duration (**B**) of ingestion events exhibited by cotton fleahopper nymphs on ThryvOn and non-ThryvOn cotton. Asterisks indicate treatments are significantly different (ANOVA, Student’s *p* < 0.05), ns indicates no significant differences.

Ingestion events were rhythmic in nature, with discernable patterns that had a definitive beginning and end. Occasionally, an ingestion event would be interrupted by a cell rupturing event but the transition from one behavioral pattern to another was distinct. Comparing the mean duration of ingestion events (Figure 5A), cotton fleahoppers spent significantly more time ingesting plant fluids on non-ThryvOn squares (914.2 ± 134.7 s, ±SEM) than on ThryvOn squares (509.2 ± 147.7 s, ±SEM) (*t*_73_ = 2.026, *p* = 0.0465). However, the mean total ingestion times per insect on ThryvOn and non-ThryvOn were not statistically different (*t*_73_ = 0.9773, *p* = 0.3316). The mean ± SEM total duration of ingestion on the non-ThryvOn squares was 3322 ± 376.2 s, while the mean ± SEM total ingestion duration on ThryvOn squares was 2776 ± 412.6 s (Figure 5B).

### 3.6. Percent Sustained Ingestion Events

**Figure 6:**
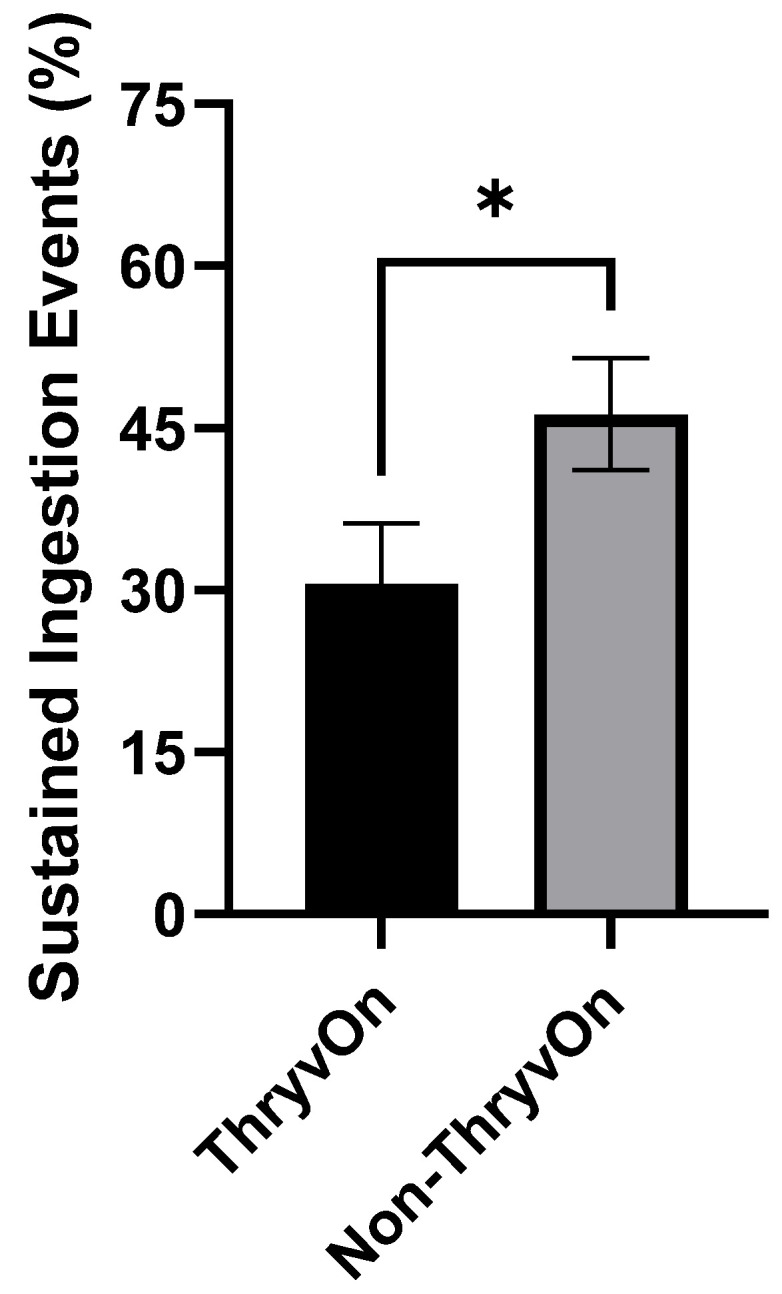
Mean ± SEM percentage of sustained ingestion events exhibited by cotton fleahopper nymphs on ThryvOn and non-ThryvOn cotton. Asterisks indicate treatments are significantly different (ANOVA, Chi-square *p* < 0.05).

A sustained ingestion event has been described as a single ingestion event uninterrupted for greater than 600 s [39]. Based on this threshold, the mean ± SEM percentage of sustained ingestion events per insect among all ingestion events per insect for non-ThryvOn was 46.3 ± 5.13% (±SEM) and 30.6 ± 5.64% (±SEM) on ThryvOn. When compared utilizing a Chi-square test, they were determined to be significantly different (*χ*^2^_1_ = 4.922, *p* = 0.0265) (Figure 6).

### 3.7. Total Duration of Non-Probing Activities

**Figure 7:**
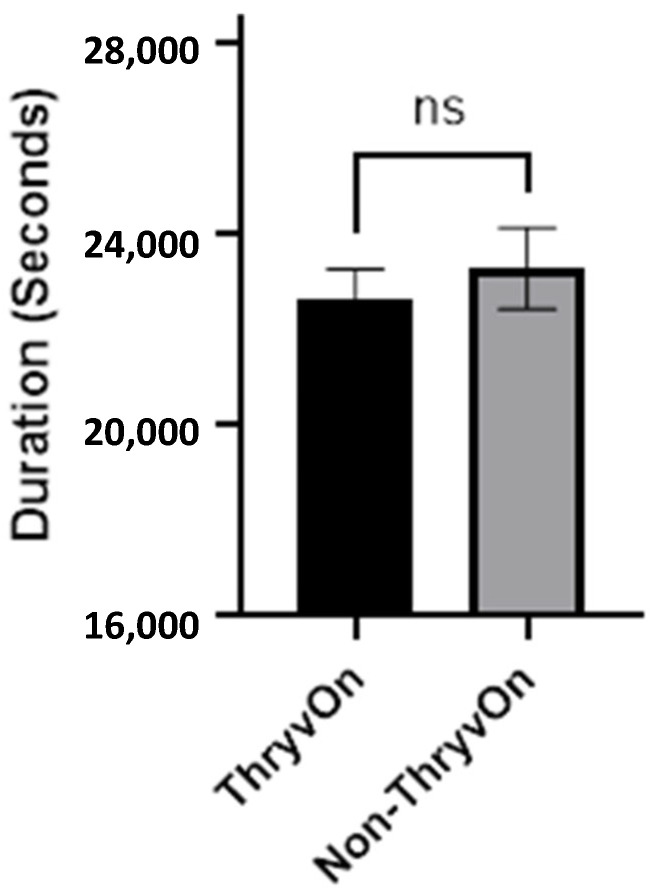
Mean ± SEM percentage of total duration of non-probing activity per insect exhibited by cotton fleahopper nymphs on ThryvOn and non-ThryvOn cotton. Asterisks indicate treatments are significantly different (ANOVA, Student’s *p* < 0.05), ns indicates no significant differences.

Since the primary objective was to determine difference in the feeding behaviors based on the presence of the Mpp51Aa2 protein, the non-probing activities such as walking and standing were summed together, as shown in Figure 7. The total duration of non-probing activity per insect on non-ThryvOn was 23279 ± 852.0 s (±SEM) and 22659.5 ± 607.9 (±SEM) on ThryvOn. There were no significant differences between the total time of non-probing activity per insect on the Non-ThryvOn and ThryvOn squares (*t*_73_ = 0.570, *p* = 0.5706).

## 4. Discussion

Our findings closely reflect the findings from Cervantes et al. [30], where both showed significantly higher numbers of cell rupturing events per insect on ThryvOn squares compared to non-ThryvOn squares, but similar numbers of ingestion events per insect. This suggests that despite the presence of the Mpp51Aa2 protein, cotton fleahoppers continued to probe and attempted to feed on ThryvOn squares. Other similar types of feeding assays with thrips and mirids have demonstrated the presence of the Mpp51Aa2 protein deters feeding, both in artificial diet and when incorporated into cotton [40,41]. In our study, the continued probing activity of cotton fleahopper nymphs on ThryvOn squares confirms that the observed feeding deterrence is likely driven by factors related to the ingestion of the protein rather than through olfactatory signals. The higher numbers of cell rupturing and ingestion events per insect can be explained in part by the significant differences in mean duration of an ingestion event. Cotton fleahoppers feeding on non-ThryvOn squares sustained longer ingestion events and therefore satisfied feeding requirements. In comparison, ingestion events on ThryvOn squares were not sustained, but nymphs attempted to feed more often. While other studies have suggested that differences in mean ingestion time while feeding on ThryvOn versus non-ThryvOn cultivars were due to differences in palatability [30], we propose an additive explanation for these feeding differences. As mentioned, the Mpp51Aa2 protein has shown activity synonymous with that of other *Bt* Cry proteins where the disruption of the insect’s midgut occurs after ingestion [11]. Ingestion of the Mpp51Aa2 protein during the earlier ingestion events on ThryvOn squares would have resulted in damage to the insect’s epithelial cells of the gut, thereby limiting subsequent ingestion events. This theory is supported by our finding as well as those of Cervantes et al. [30], wherein ingestion events would still occur on ThryvOn cotton but were prematurely terminated due to the insects’ inability to sustain ingestion.

The slope of the regression model of cell rupturing events per Insect against ingestion events per insect was 0.3208 with a 95% confidence interval of 0.263 to 0.379 on non-ThryvOn squares, inferring that approximately every third cell rupturing event was accompanied by an ingestion event (Figure 3). Although a logistic growth model best described nymph feeding behavior on ThryvOn squares, the general trend of the logistics growth was similar to the linear regression of the non-ThryvOn until the maximum number of ingestion events per insect on ThryvOn plateaued at 6.68 events. The plateau suggests that the ingested Mpp51Aa2 protein arrested further ingestion events. Jerga et al. [11] demonstrated that when tarnished plant bug nymphs consumed the Mpp51Aa2 protein, significant damage and cellular sloughing were observed in the midgut epithelium. Likewise, damage to the insect’s midgut from the Mpp51Aa2 protein offers a potential explanation as to why the ingestion events of cotton fleahopper nymphs on ThryvOn squares failed to continue.

Historically, salivation during mirid feeding is known to cause production of ethylene in cotton, resulting in fruit abscission [18,21,22,24]. Therefore, it can be postulated the increase in fruit retention observed on ThryvOn cotton during field trials and caged studies [13,14,15,16,17] was due to suppressed feeding and salivating behaviors. However, our current study revealed the number of cell rupturing events per insect was numerically higher on ThryvOn squares compared to the non-ThryvOn cultivar, and there was no statistical difference in the duration of cell rupturing events between the two cotton treatments. As described by Backus et al. [27], cell rupturing behaviors of mirids such as the cotton fleahopper include multiple actions including proboscis insertion, salivation, taste, and the transitional periods between each action. Waveforms of each component of cell rupturing are difficult to delineate but it is possible that the duration of salivation was reduced as probing events continued. If nymphs had salivated after the initiation of a probe, the duration of the subsequent cell rupturing events could be composed mostly of time spent tasting and attempting to transition to ingestion. However, our findings suggest that nymphs failed to transition from cell rupturing to ingestion, likely due to disruption of the insect’s gut by the Mpp51Aa2 protein.

## 5. Conclusions

In summary, monitoring cotton fleahopper feeding with electropenetrography offers valuable insights into the insects’ feeding behaviors on cotton squares. Patterns in feeding waveforms produced by cotton fleahopper feeding were similar to the stereotypical waveform patterns produced by *Lygus* spp. [27,29]. Therefore, utilizing documented waveforms, cotton fleahopper nymph feeding behaviors were classified and the duration of the behaviors was quantified for comparison to determine the activity of the Mpp51Aa2 protein expressed in ThryvOn cotton on cotton fleahopper nymph feeding. While comparing the feeding behaviors of cotton fleahopper nymphs on ThryvOn and non-ThryvOn squares, differences were noted in the number of cell rupturing events, the mean duration of ingestion events, and the percentage of ingestion events that were sustained for more than 10 min. Collectively, our findings indicate the Mpp51Aa2 protein affects the feeding behavior of cotton fleahopper nymphs. While the mechanism responsible for the change in cotton fleahopper feeding behavior associated with the Mpp51Aa2 protein is not fully understood, our results in combination with other studies suggest it may be related to the disruption of the insect’s gut. Regardless, our study provides evidence of ThryvOn cotton’s effect on cotton fleahoppers. Thus, utilizing ThryvOn cotton as part of an insect management system should result in increased fruit retention and potentially reduce the need for foliar insecticide applications for managing small and moderately cotton fleahoppers populations.

## Data Availability

The raw data supporting the conclusions of this article will be made available by the authors on request.
